# The Role of Sirtuin-1 Isoforms in Regulating Mitochondrial Function

**DOI:** 10.3390/cimb46080522

**Published:** 2024-08-14

**Authors:** Pankaj Patyal, Fathima S. Ameer, Ambika Verma, Xiaomin Zhang, Gohar Azhar, Jyotsna Shrivastava, Shakshi Sharma, Rachel Zhang, Jeanne Y. Wei

**Affiliations:** Donald W. Reynolds Department of Geriatrics and Institute on Aging, University of Arkansas for Medical Sciences, Little Rock, AR 72205, USA; ppatyal@uams.edu (P.P.); suraiyaameer83@gmail.com (F.S.A.); averma@uams.edu (A.V.); zhangxiaomin@uams.edu (X.Z.); azhargohar@uams.edu (G.A.); jshrivastava@uams.edu (J.S.); ssharma@uams.edu (S.S.); rz2714@cumc.columbia.edu (R.Z.)

**Keywords:** sirtuin-1, domain loss, mitochondria, bioenergetics, histone-H4, acetylation

## Abstract

The sirtuin-1 (SIRT1) gene contains multiple exons that usually undergo alternative splicing. The exclusion of one or more exons causes domain loss in the alternatively spliced isoforms and may change their functions. However, it is not completely established to what extent the loss of a non-catalytic domain could affect its regulatory function. Using muscle cells and SIRT1-knockout cells, we examined the function of the constitutively spliced isoform (SIRT1-v1) versus the alternatively spliced isoforms SIRT1-v2 and SIRT1-v3 that had lost part of the N-terminal region. Our data indicate that partial loss of the N-terminal domains in SIRT1-v2 and SIRT1-v3 attenuated their function. The full-length SIRT1-v1 significantly increased the oxidative phosphorylation and ATP production rate. Furthermore, SIRT1-v1 specifically upregulated the mitochondrial respiratory complex I without affecting the activity of complexes II, III, and IV. Additionally, domain loss affected the regulation of site-specific lysine acetylation in the histone H4 protein, the gene expression of respiratory complex I subunits, and the metabolic balance of oxidative phosphorylation versus glycolysis. Since alternatively spliced isoforms tend to increase with advancing age, the impact of SIRT1 isoforms on mitochondrial respiratory complexes warrants further investigation.

## 1. Introduction

The alternative splicing process often enables eukaryotic cells to generate multiple mRNA transcripts from a single gene, which dramatically increases the transcriptomic and proteomic diversity in the entire genome [1,2]. During this process, one or more exons may be skipped/excluded while the remaining exons from the same gene link together with different combinations, which generate multiple mature mRNA transcripts. Most alternatively spliced isoforms may lack one or more exons versus their full-length isoform and may lose part of the structural or functional domain. The skipped exon(s) may encode the regulatory, binding, or catalytic domains [3]. As a result, the alternatively spliced isoforms may have a distinct structural and/or biological function, increasing gene expression and cellular function complexity [1,4]. The proportion of the isoforms in each cell or tissue may change during various stages of development, maturation, and aging, but also change in response to physiological and pathological stimuli [5,6,7,8].

The sirtuin-1 (SIRT1) protein is an NAD^+^-dependent deacetylase that regulates cellular function through deacetylation of its target proteins. SIRT1 deacetylates the histone proteins and changes the chromatin architecture and structure that control transcriptional regulation and gene expression [9,10,11]. SIRT1 also regulates the signaling pathways leading to mitochondrial biogenesis, mitochondrial gene expression, and mitochondrial function [12,13]. However, it has not been completely explored whether SIRT1 specifically targets one or more of the mitochondrial respiratory complexes, and whether the non-catalytic domain (e.g., the N-terminal domain) is required to maintain its regulatory function. The SIRT1 gene is a multi-exon gene that undergoes alternative splicing [14,15]. Exon-skipping has been observed in the alternatively spliced isoforms, which generates shorter isoforms that lack one or more exons [1]. Here, we examined the alternatively spliced isoforms SIRT1-v2 and SIRT1-v3 versus the constitutively spliced isoform (SIRT1-v1). The v2 and v3 isoforms have lost part of the N-terminal region that includes both the NLS1 and NLS2 nuclear localization sequences. These truncated SIRT1 isoforms are ideal tools to analyze the cellular function associated with domain losses. The data indicate that SIRT1-v1 significantly increases mitochondrial oxidative phosphorylation and especially upregulates mitochondrial respiratory complex I, without significantly affecting complexes II, III, and IV, whereas loss of the N-terminal region in v2 and v3 attenuates their function. The data indicate that partial domain loss in v2 and v3 attenuates their function in the regulation of mitochondrial oxidative phosphorylation, site-specific lysine acetylation, and mitochondrial gene expression.

## 2. Materials and Methods

### 2.1. Cell Culture and SIRT1 Plasmid Constructs

The DMEM medium, fetal bovine serum, and Lipofectamine 2000 were purchased from Thermo Fisher Scientific (Carlsbad, CA, USA). The C2C12 cell line (CRL-1772) and HEK293T cell line (CRL-3216) were purchased from ATCC (Manassas, VA, USA) [16]. The HEK293T SIRT1-knockout cell line (HEK293T SIRT1 KO) was obtained from Kerafast, Inc., Boston, MA, USA [17]. All the cells were cultured according to the provider’s instructions and as previously described [18].

The GFP gene fragment was fused in-frame with the N-terminus of SIRT1 isoform v1, v2, and v3 genes, and subcloned into pReceived-M29 expression plasmid vectors (Genecopoeia, Rockville, MD, USA), respectively [19]. These three SIRT1 isoform expression constructs were sequenced at the UAMS (University of Arkansas for Medical Sciences) sequence core facility, which confirmed that the SIRT1 isoform inserts within the expression vectors matched perfectly the sequences in the NCBI database. SIRT1-v1 matched with NM_012238, SIRT1-v2 matched with NM_001142498, and SIRT1-v3 matched with NM_001314049, respectively.

### 2.2. Transfections

**Figure 1:**
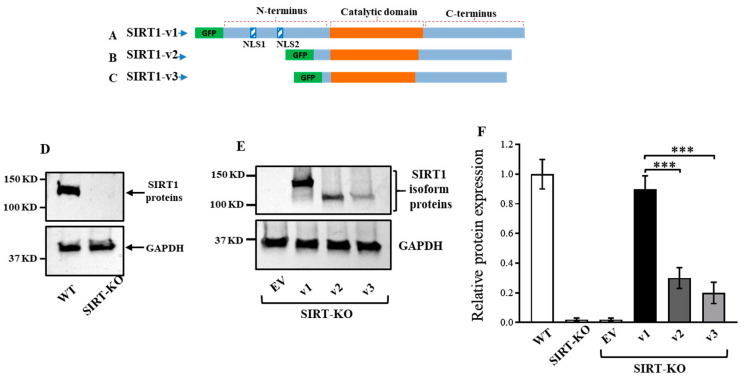
Three expression constructs containing GFP-SIRT1 isoforms. The v1 coding region sequence is 747 amino acids (AAs) (**A**), the v2 coding region sequence is 452 AAs (**B**), and the v3 coding region is 444 AAs (**C**). The SIRT1 protein expression was present in wild-type cells, but absent in SIRT1-KO cells (**D**). The SIRT1 isoform proteins were detected in the SIRT1-KO cells after transfection of empty vector (EV) and SIRT1 isoform plasmid vectors (**E**). The relative protein levels of SIRT1 isoform proteins are shown in (**F**). Data are mean ± SD of three repeats. Significant difference *** *p* < 0.001 by one-way ANOVA with Tukey’s procedure.

Plasmid DNA transfections were performed using Lipofectamine 2000 (Invitrogen, Carlsbad, CA, USA) according to the manufacturer’s instructions. Fifty microliters of cell culture medium without fetal bovine serum was mixed with 2 μL of Lipofectamine. Equal and appropriate DNA (µg) was given to 50 μL of cell culture medium without fetal bovine serum to control and experimental groups. The two solutions were mixed and incubated for 20 min at room temperature. The cells were treated with the mixture at the appropriate dilution. The transfection efficiency was determined microscopically by counting GFP-colored cells and western blot analysis was also performed to check the overall transfection of SIRT1 isoform plasmids. The western blot analysis of total SIRT1 protein shown in Figure 1E demonstrates the transfection efficiency of SIRT1 isoform plasmids in SIRT1 KO HEK293T cells, whereas representative western blots in Supplementary Figure S1 show the transfection efficiency of SIRT1 plasmids in C2C12 cells.

### 2.3. Analysis of Mitochondrial Membrane Potential

The Image-iT™ TMRM reagent (Thermo Fisher, Waltham, MA, USA, cat#I34361) was used to measure the mitochondrial membrane potential in the live cells, and MitoTracker Red CMXRos (Thermo Fisher, cat# M7512) was used to measure the mitochondrial signal intensity in fixed cells [20,21,22,23]. The C2C12 cells were seeded in a 35 mm MatTek microscopy dish with a glass bottom (Thermo Fisher, cat. # NC9574048) and grown in DMEM supplemented with 10% FBS (fetal bovine serum) at 37 °C and 5% CO_2_ overnight. The expression plasmid constructs containing the empty vector and the SIRT1 isoforms (v1, v2 v3) were transfected into the cells using Lipofectamine 2000. At 24 h after the transfection, the cells were prepared for the imaging analysis. The staining with the TMRM reagent was performed according to the manufacturer’s instruction manual.

The staining with MitoTracker™ Red CMXRos was performed as previously described [18,20]. Briefly, at 24 h after the transfection, the cells were prepared for the imaging analysis. The cells were fixed with 3.7% formaldehyde (Thermo Fisher, SF100-4) for 10 min and permeabilized with 0.2% Triton X-100 in PBS buffer for 5 min. The cells were rinsed with PBS buffer and blocked with 3% BSA in PBS buffer for 1 h. The cells were stained with MitoTracker™ Red CMXRos (Thermo Fisher, Waltham, MA, cat# M7512) for 30 min as previously described [20]. The cell nuclei were stained with a 1:1000 solution of DAPI (Thermo Fisher, Waltham, MA, USA, D1306) for 5 min, then microscopic images were analyzed and recorded with a Zeiss LSM 880 confocal microscope with the ZEN blue 3.2 software (Carl Zeiss Microscopy, White Plains, NY, USA). The collected images were analyzed, and the fluorescence intensity was quantified using the ImageJ software (Version 1.54g, National Institutes of Health) [18].

### 2.4. Determination of Mitochondrial Respiration Rate, Glycolytic Rate, and ATP Production Rate

The measurement of the mitochondrial respiration rate, glycolytic rate, and ATP production rate was performed using an Agilent XFe96 Analyzer and reagents (Seahorse Bioscience, Billerica, MA, USA) as previously described [18]. Briefly, the C2C12, HEK293T, and HEK293T SIRT1-KO cells were seeded at 15,000 cells/well in Seahorse XF 96 well cell culture plates. The cells were transfected with GFP-empty plasmid or the plasmid containing the GFP-SIRT1-v1, v2, or v3 fusion gene for 24 h. The real-time cell metabolic function was measured in a Seahorse XFe96 Analyzer using the XF assay kits, reagents, and cell assay media, including XF Cell Mito Stress Test Kit (Part Number:103015-100), XF Glycolytic Rate Assay Kit (Part Number:103344-100), and XF Real-Time ATP Rate Assay Kit (Part Number:103592-100). Data are normalized to equal number of cells in all variables by performing a cell count using the trypan blue exclusion method.

### 2.5. Assessing the Mitochondrial Electron Transport Chain Activity in Intact Cells Using Oroboros High-Resolution Respirometry

The Oroboros oxygraph-O2K high-resolution respirometer (Oroboros Instruments GmbH, Innsbruck, Austria) was used to analyze the mitochondrial bioenergetics in intact C2C12 cells (1 × 10^6^/sample) as previously described [24]. The OCR (oxygen consumption rate) in the complexes was further examined according to the substrate–inhibitor–titration protocol, as described in [24]. Briefly, 2 × 10^6^ cells were incubated in digitonin (Sigma, Milwaukee, WI, USA; D5628; 8 μM/million cells) and prepared in MiRO5 buffer for 20 min at 4 °C to permeabilize the cells. The DatLab 6.2 software (Innsbruck, Austria) was used to perform the data analysis; the OCR of intact cells and from each mitochondrial complex was expressed as oxygen flux (pmol/s*million cells).

### 2.6. Quantitative Reverse-Transcriptase PCR

The reverse transcription and RT-PCR reagents were purchased from Applied Biosystems (Waltham, MA, USA). The PCR primers were designed and selected using real-time PCR assay design software from both IDT (Integrated DNA Technologies) and PrimerBlast (NCBI). All the primers were synthesized by Integrated DNA Technologies (Coralville, Iowa) [25]. The quantitative RT-PCR amplification was performed in a QuantStudio 3 Real-Time PCR System (Applied Biosystems, Waltham, MA, USA). The primer sequences were NDUFAB1 forward acattgcagataagaaggatgtgt, reverse tcactcttggttgtcagtgtgt. NDUFS1 forward caggggctgcttacacagaa, reverse aggcgtcactgctactttgg. NDUFV1 forward aaagccatcgctcgtctcat, reverse ttcatccagtcaacgccctc. NDUFV2 forward tcggtttgcttattcccacct, reverse gaccgagatagtggtcctgt. NDUFA1 forward gtccactgcgtacatccaca, reverse acgtctatcgcgttccatca. NDUFA5 forward acgagaagctggatatggtca, reverse acttcaccaccctgaagcaag. NDUFA10 forward gctgaaacaggacgactgga, reverse ggtagaccggaatggtcgtg. NDUFA12 forward ggatgtggatggaagcatgg, reverse tggattagtcgtcggagggt. NDUFB10 forward gcagacctcgctccctaac, reverse cttggcatgctgtcgttcaa. NDUFB11 forward ccagaacccgaggacgaaaa, reverse cacgaaggtggtcccaaaga. NDUFC2 forward tggacaacatgctgcggat, reverse tccagcaaagacaaaggacg. Relative expression values were obtained by normalizing CT values of the mRNA genes in comparison with CT values of the endogenous control (5S RNA) using the 2^–ΔΔCt^ method [18,25]. The primers used in the current study were designed with the SciTools Web software, IDT (Version 2.2.3), and ordered from IDT (Integrated DNA Technologies, Inc., San Diego, CA, USA). The minimum information for publication of quantitative real-time PCR experiments (MIQE) guidelines were adhered to in the current study [26].

### 2.7. Immunoprecipitation and Western Blot Analysis

The immunoprecipitation and western blotting were performed as previously described [27,28]. Briefly, C2C12 cells were collected after 24 h of transfection, washed twice with cold PBS, and lysed in RIPA lysis buffer system (Santa Cruz, Dallas, TX, USA, sc-24948A) for 10 min at 4 °C. The protein concentration was measured using a Pierce™ BCA Protein Assay Kit (Thermo Fisher, 23225). The membrane was incubated with the indicated antibodies and detected by using a chemiluminescence method. For immunoprecipitation, total cell lysates were incubated with appropriate antibodies overnight, and subsequently, rotated with protein A/G beads for 2–4 h at 4 °C. The beads were washed three times using RIPA lysis buffer, mixed with 2 × SDS sample buffer, and boiled for 5–10 min. The co-precipitates were analyzed by western blot analysis. The antibodies that were used included PGC-1α (1:1000, Santa Cruz, sc-518025), histone H4 (1:1000, Cell signaling, L64C1), anti-acetyl-histone H4 (1:1000, Lys16) (Cell signaling, E2B8W), anti-acetyl-histone H4 (1:1000, Lys12) (Cell signaling, D2W6O), anti-acetyl-histone H4 (1:1000, Lys5) (Cell signaling, D12B3), anti-acetyl-histone H4 (1:1000, Lys8) (Cell signaling, 2594), GAPDH (1:5000, Santa Cruz, sc-365062) and β-actin (1:5000, Santa Cruz, sc-47778). The secondary antibodies used were anti-mouse HRP (1:5000, Invitrogen, Carlsbad, CA, USA; 62-6520), anti-goat HRP (1:5000, Santa Cruz, Dallas, TX, USA; sc-20200), and anti-rabbit AP (1:5000, Bio-Rad, Hercules, CA, USA; 64251130). Immunoreactive bands were visualized with ECL and iBright™ CL1500 (Invitrogen) was used for imaging.

### 2.8. Statistical Analysis

Data are given as mean value ± SD, with n denoting the number of experiments unless otherwise indicated. A two-tailed *t*-test was used to determine the differences between the two groups. One-way analysis of variance (ANOVA) was adopted to analyze the differences among groups by using the Tukey’s procedure with the GraphPad Prism 9.3.1 software. A *p*-value of less than 0.05 was considered as statistically significant.

## 3. Results

### 3.1. The SIRT1 Isoforms and Their Impact on Mitochondrial Membrane Potential

The cDNA fragments containing SIRT1-v1, SIRT1-v2, and SIRT1-v3 were subcloned into expression vectors, and the isoform coding region sequences were 747 amino acids (AAs) for v1, 452 AAs for v2, and 444 AAs for v3 (Figure 1A–C). The alternative splicing resulted in the loss of nuclear localization signal 1 (NLS1) and NLS2 in both the v2 and v3 isoforms (Figure 1B,C). The green fluorescent protein (GFP) was fused in-frame with the N-terminus of the SIRT1 isoform proteins (Figure 1A–C). To measure the protein expression of the SIRT1 isoforms, the expression vectors containing the v1, v2, and v3 isoforms were transfected into the HEK293T (wild-type) and HEK293T-knockout (SIRT1-KO) cells.

A SIRT1 protein band was detected in the wild-type HEK293T cells but not detected in the SIRT1-KO cells (Figure 1D). After the SIRT1-KO cells were transfected with the v1, v2, and v3 isoform expression vectors, the corresponding v1, v2, and v3 isoform proteins were detected in the cell lysate (Figure 1E,F), indicating that all three isoform proteins were expressed from the transfected vectors.

**Figure 2:**
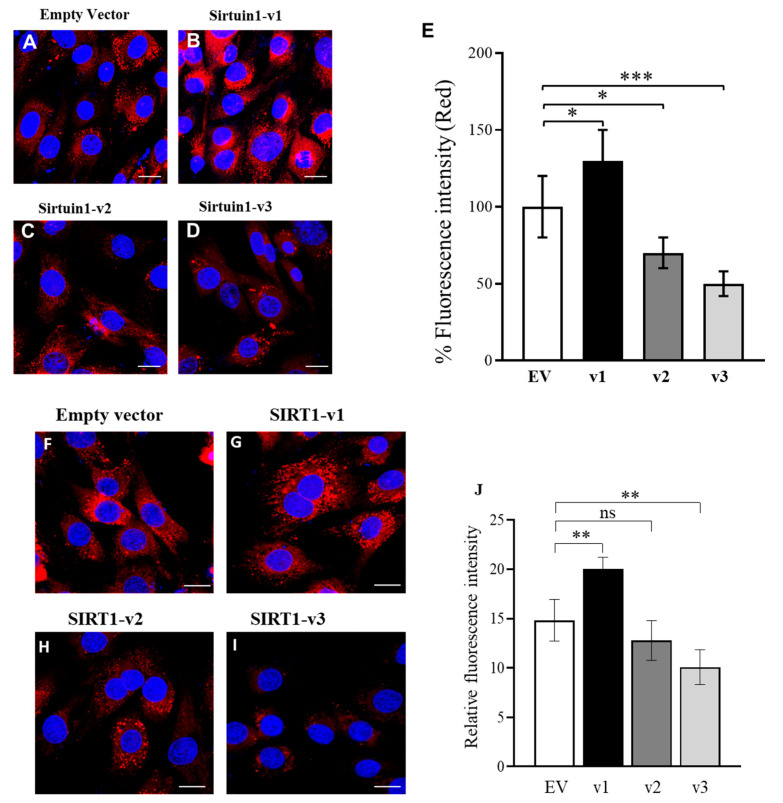
The mitochondrial membrane potential (MMP) and the mitochondrial mass and content. The SIRT1 isoform constructs were transfected into C2C12 cells. At 24 h after transfection, the cells were stained with the Image-iT™ TMRM reagent (**A**–**E**) for the measurement of mitochondrial membrane potential (MMP), or MitoTracker (red) conjugated dye for the detection of the mitochondrial mass and content (**F**–**J**). The DAPI (blue) was added for nuclear counterstaining. Empty vector (**A**); SIRT1-v1 increased the fluorescent signal intensity, indicating the elevation of mitochondrial membrane potential (MMP) ((**B**,**E**), * *p* ≤ 0.01); SIRT-v2 reduced MMP ((**C**,**E**), * *p* ≤ 0.01); SIRT1-v3 reduced MMP ((**D**,**E**), ** *p* ≤ 0.01). The stained cells were randomly selected from microscopic fields in four individual repetitions where the fluorescence intensity was quantified using ImageJ. The fluorescent intensity of clearly identifiable mitochondria in randomly selected 80–90 cells per experiment were measured. Significant difference (*n* = 4, ns, *p* > 0.05 * *p* ≤ 0.05; ** *p* ≤ 0.01, *** *p* < 0.001) between EV and SIRT1 isoforms by one-way ANOVA with Tukey’s procedure. Zeiss Plan Apo 63× oil objective is used; scale bars indicate 20 μm.

To measure the mitochondrial membrane potential (MMP), tetramethylrhodamine, methyl ester (TMRM) was used to stain the live cells and the fluorescent signal intensity was detected by confocal microscopy (Figure 2A–E). The data indicate that the SIRT1-v1 isoform significantly increased the mitochondrial membrane potential (MMP), while v2 and v3 reduced the MMP in the C2C12 muscle cells. In addition, after staining with MitoTracker Red, confocal microscopy was used to measure the mitochondrial mass and content [21,29]. The SIRT1-v1 isoform significantly upregulated the mitochondrial mass and content (Figure 2F,J), while the SIRT1-v2 isoform had no effect (Figure 2H,J), but SIRT1-v3 reduced the mitochondrial mass and content (Figure 2I,J).

To measure the mitochondrial membrane potential (MMP), the Image-iT™ TMRM reagent was used to stain the live cells and the fluorescent signal intensity was detected by confocal microscopy (Figure 2A–E). TMRM (tetramethylrhodamine, methyl ester) is a cell-permeant dye that accumulates in active mitochondria with intact membrane potentials, which has been considered as a mitochondrial membrane potential indicator. The data indicate that the SIRT1-v1 isoform significantly increased the mitochondrial membrane potential (MMP), while v2 and v3 reduced the MMP in the muscle cells. In addition, after staining with MitoTracker Red, confocal microscopy was used to measure the mitochondrial mass and content [22]. The SIRT1-v1 isoform significantly upregulated the mitochondrial mass and content (Figure 2F,J), while the SIRT1-v2 isoform had no effect (Figure 2H,J), but SIRT1-v3 reduced the mitochondrial mass and content (Figure 2I,J).

### 3.2. Loss of N-Terminal Region in SIRT1 Isoforms Affected the Regulation of Cellular Metabolism

**Figure 3:**
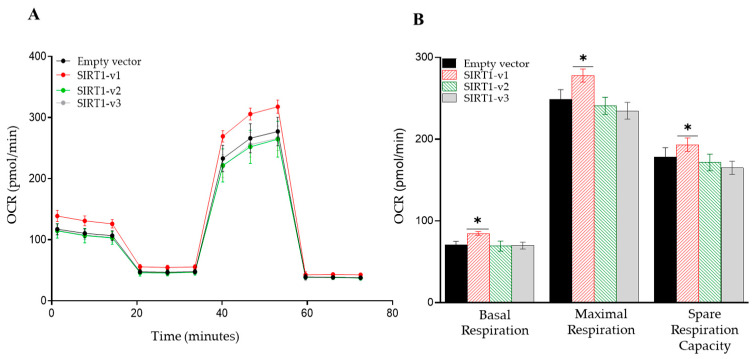

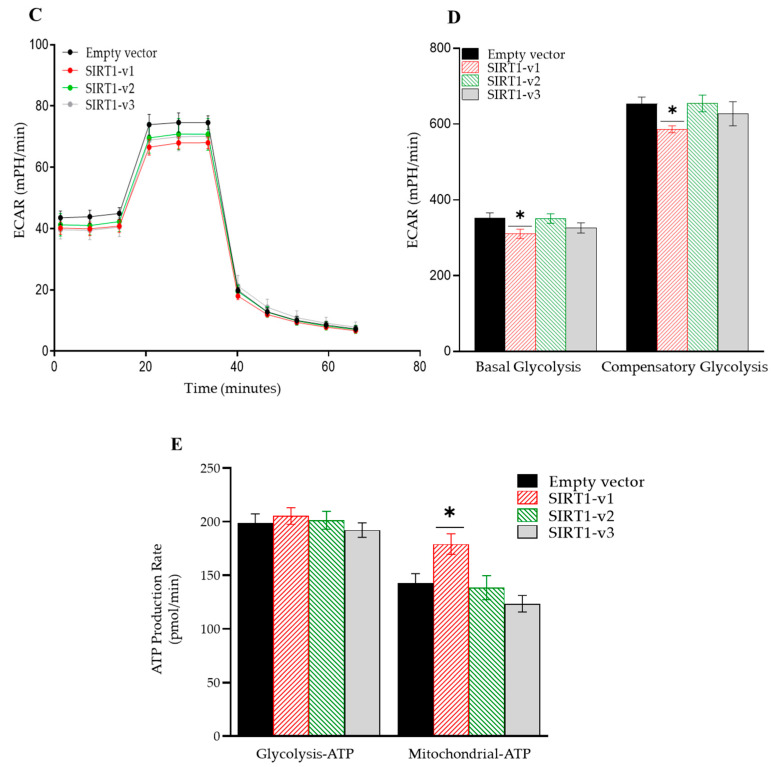
Analysis of the effect of SIRT1 isoforms on mitochondrial function with the Seahorse XFe96 analyzer. Mitochondrial respiratory profile of SIRT1 isoforms. (**A**) Oxygen consumption rate (OCR) measurements were obtained over time (min) using an extracellular flux analyzer, Seahorse XFe96. (**B**) Basal mitochondrial OCR of SIRT1 isoform-transfected C2C12 cells was derived by subtracting non-mitochondrial OCR. Maximum OCR was stimulated by FCCP addition. The spare respiratory capacity was calculated as the difference between maximal and basal OCR. (**C**) Glycolytic rate (ECAR) of SIRT1 isoform-transfected C2C12 cells. (**D**) A quantitative analysis of the basal and compensatory glycolysis. (**E**) Quantification of total ATP production rate of C2C12 cells transfected with different SIRT1 isoforms. Graphs represent data normalized with equal cell count, *n* = 3, one-way ANOVA, Tukey’s post hoc test (* *p* < 0.05).

The mitochondrial oxygen consumption rate (OCR), glycolysis rate (ECAR), and ATP production rate were used as indicators to evaluate the impact of the SIRT1 isoforms on energy metabolism and overall cellular function. Here, the Seahorse XFe96 Analyzer was used to measure the mitochondrial oxygen consumption rate and glycolytic rate at 24 h after the transfection of the myocytes with the SIRT1-v1, v2, and v3 isoform expression vectors. Compared to an empty vector (EV), the SIRT1-v1 isoform significantly increased the basal oxygen consumption (*p* < 0.05, *n* = 3), and significantly increased the maximal respiration (*p* < 0.05, *n* = 3) and spare respiration capacity (*p* < 0.05, *n* = 3) (Figure 3A,B). However, isoform SIRT1-v2 did not change the basal respiration, maximal respiration, or spare respiration capacity (Figure 3A,B). Isoform SIRT1-v3 also did not change the basal respiration, but it slightly reduced both the maximal respiration and spare respiration capacity (Figure 3A,B).

The XFe96 Analyzer was also used to quantify the cellular ATP level after the transfection of the SIRT1 isoform vectors. Determined by ATP real-time rate assay, the SIRT1-v1 expression significantly increased the mitochondrial-ATP production rate (*p* < 0.05, *n* = 3) (Figure 3E). SIRT1-v2 did not change the mitochondrial-ATP production rate (Figure 3E). SIRT1-v3 slightly reduced the mitochondrial-ATP production rate (Figure 3E). None of the three isoforms significantly changed the glycolysis-ATP production rate (Figure 3E).

Additionally, the SIRT1-v1 isoform significantly reduced the basal glycolysis (*p* < 0.05, *n* = 3) and the compensatory glycolysis (*p* < 0.05, *n* = 3) (Figure 3C,D). Isoform v2 did not change the basal glycolysis (Figure 3C,D) or the compensatory glycolysis (Figure 3C,D). Isoform v3 did not change the basal glycolysis or the compensatory glycolysis either (Figure 3C,D).

The XFe96 Analyzer was also used to quantify the cellular ATP level after the transfection of the SIRT1 isoform vectors. Determined by ATP real-time rate assay, SIRT1-v1 expression significantly increased the mitochondrial-ATP production rate (*p* < 0.05, *n* = 3) (Figure 3E). SIRT1-v2 did not change the mitochondrial-ATP production rate (Figure 3E). SIRT1-v3 slightly reduced the mitochondrial-ATP production rate (Figure 3E). None of the three isoforms significantly changed the glycolysis-ATP production rate (Figure 3E).

### 3.3. Loss of N-Terminal Region in SIRT1 Isoforms Affected the Regulation of Mitochondrial Respiratory Complex I

**Figure 4:**
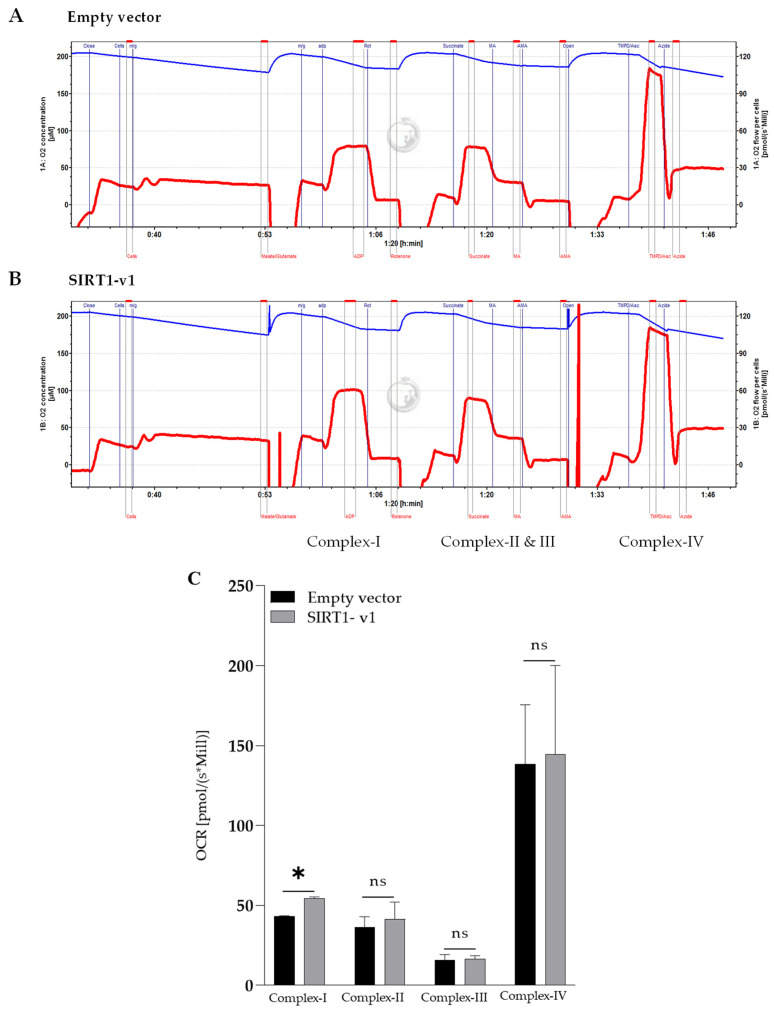
Assessment of the activity of mitochondrial electron transport chain complexes I, II, III, and IV using the Oroboros high-resolution respirometer. High-resolution respiratory analysis of oxidative phosphorylation at mitochondrial complexes. The C2C12 cells transfected with SIRT1 isoforms were permeabilized and were used to determine OCR level at complexes of ETC. Representative traces of the high-resolution respirometer were obtained using a multiple substrate–inhibitor–titration protocol. (**A**) Representative trace of oxygraph-O2K for empty vector transfection. (**B**) Representative trace of high resolution respiratory for SIRT1-v1 transfection. (**C**) Quantification of complexes activity shows the OCR at complex I of ETC was specifically increased with SIRT1-v1, but no change was observed at other complexes (II–IV). Significant difference (ns, *p* > 0.05 * *p* < 0.05, *n* = 3), analyzed by a two-tailed Student’s *t*-test.

Mitochondrial cellular respiration and ATP production involves five respiratory complexes [30,31]. The OCR, ECAR, and ATP rate data from the XFe96 Analyzer measurement reflect the overall mitochondrial function, which involves the various ETC complexes. To determine whether SIRT1 isoforms would target specific components of the mitochondrial respiration chain, the Oroboros oxygraph-O2K high-resolution respirometer was employed to assess the activities of mitochondrial complexes I, II, III, and IV [24]. As shown in Figure 4, SIRT1-v1 significantly upregulated mitochondrial complex I (*p* < 0.05, *n* = 3), but did not significantly change the activity of complexes II, III, and IV. Whereas SIRT1-v2 and v3 did not significantly change the activity of complexes I, II, III, and IV (Figures S2 and S3). The data indicate that SIRT1-v1 specifically regulated complex I. In addition, loss of part of the SIRT1 gene in the N-terminus significantly attenuated the function of the SIRT1 gene in the regulation of complex I. The data indicate that the full-length SIRT1 coding region was required for the positive regulation of mitochondrial respiratory complex I, while exclusion of the N-terminus resulted in the loss of this function.

### 3.4. Loss of N-Terminal Region in SIRT1 Isoforms Affected the Regulation of Mitochondrial Genes and Histone H4 Acetylation

Since the experimental data indicated that SIRT1 isoforms modulated cellular respiration, and mainly affected the mitochondrial complex 1 function, we next analyzed the gene expression of complex I subunits, which included NDUFAB1, NDUFS1, NDUFV1, NDUFV2, NDUFA1, NDUFA5, NDUFA10, NDUFA12, NDUFB10, NDUFB11, and NDUFC2.

**Figure 5:**
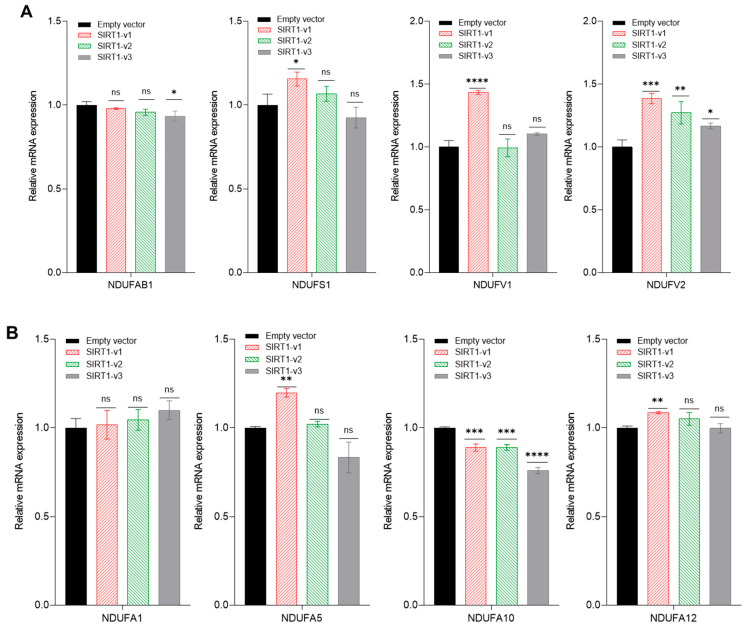

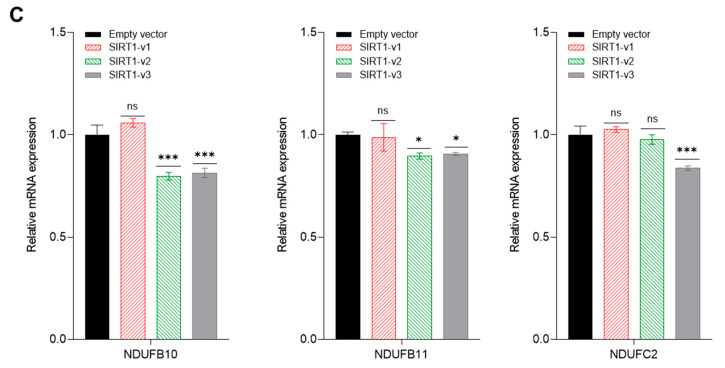
Real-time RT-PCR analysis of mitochondrial genes at complex I subunits. (**A**) qRT-PCR analysis of NDUFAB1, NDUFS1, NDUFV1, and NDUFV2 gene expression following the transfection of C2C12 cells with SIRT1 isoforms. (**B**) NDUFA1, NDUFA5, NDUFA10, and NDUFA12. (**C**) NDUFB10, NDUFB11, and NDUFC2. Significant difference (* *p* < 0.05, ** *p* < 0.01, *** *p* < 0.001, **** *p* < 0.0001, ns: *p* > 0.05, *n* = 3), analyzed by one-way ANOVA with Tukey’s procedure.

SIRT1-v1 transfection significantly upregulated the subunits NDUFS1 (*p* < 0.05, *n* = 3), NDUFV1 (*p* < 0.0001, *n* = 3), NDUFV2 (*p* < 0.001, *n* = 3), NDUFA5 (*p* < 0.01, *n* = 3), and NDUFA12 (*p* < 0.01, *n* = 3). The SIRT1-v1 isoform downregulated the subunit NDUFA10 (*p* < 0.001, *n* = 3), but did not affect the NDUFA1, NDUFA10, NDUFB11, and NDUFC2 subunits. SIRT1-v2 upregulated NDUFV2 (*p* < 0.01, *n* = 3), but downregulated NDUFV2 (*p* < 0.01, N = 3), NDUFA10 (*p* < 0.001, *n* = 3), NDUFB10 (*p* < 0.001, *n* = 3), and NDUFB11 (*p* < 0.05, *n* = 3). The SIRT1-v3 isoform upregulated NDUFV2 (*p* < 0.05, *n* = 3), but downregulated NDUFAB1 (*p* < 0.05, *n* = 3), NDUFA10 (*p* < 0.0001, *n* = 3), NDUFB10 (*p* < 0.001, *n* = 3), NDUFB11 (*p* < 0.05, *n* = 3), and NDUFC2 (*p* < 0.001, *n* = 3) (Figure 5).

**Figure 6:**
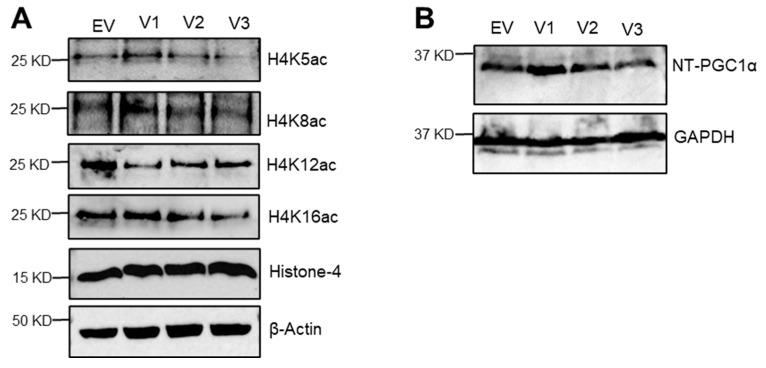
Immunoprecipitation and western blot analysis. (**A**) Twenty-four hours post-transfection with SIRT1 isoforms, C2C12 cell lysate analyzed for histone-H4 acetylation. Immunoblot represents histone-H4-acetylated lysine at K5, K8, K12, and K16, respectively. Total histone H4 protein and β-actin were used as loading controls. (**B**) SIRT1-v1 increases the NT-PGC1α protein expression; representative western blot of NT-PGC1α with different SIRT1-v1 isoforms [27,33]. GAPDH was used as a loading control. Blots are representative of three repeats.

Since SIRT1 protein regulates histone acetylation, which controls the chromatin structure and architecture, the effect of SIRT1 isoforms on the acetylation of site-specific lysine residue in histone H4 was determined. Transfection of the SIRT1 isoforms showed that the acetylation status of histone H4 K5 was slightly increased by SIRT1-v1, unchanged by SIRT1-v2, and reduced by SIRT1-v3. H4K8 acetylation was slightly increased by the isoform v1, but unchanged by v2 and v3. H4k12 acetylation was reduced by all three isoforms. H4K16 acetylation was slightly upregulated by v1, but unchanged by v2, and slightly downregulated by v3 (Figure 6). Furthermore, PGC-1α induced an increase in mitochondrial numbers and intracellular ATP concentration in a variety of cells. Rodgers et al. have shown that SIRT1 interacts with PGC-1α and deacetylates PGC-1α at specific lysine residues in an NAD^+^-dependent manner [32]. Our findings demonstrate PGC-1α expression stimulated mitochondrial biogenesis by the SIRT-v1 isoform but not by SIRT-the v2 and v3 isoforms (Figure 6).

### 3.5. The Impact of SIRT1 Isoforms on Cellular Respiration in the SIRT1-Knockout Cells

**Figure 7:**
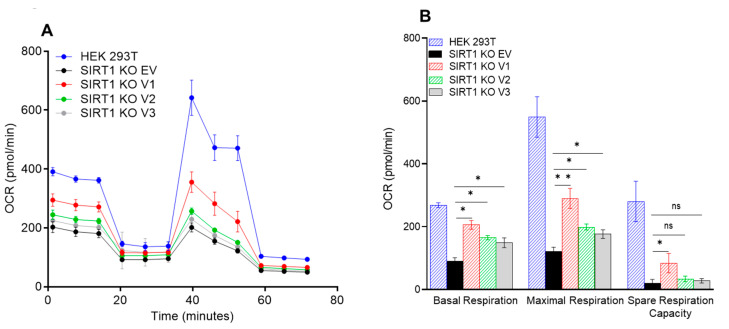

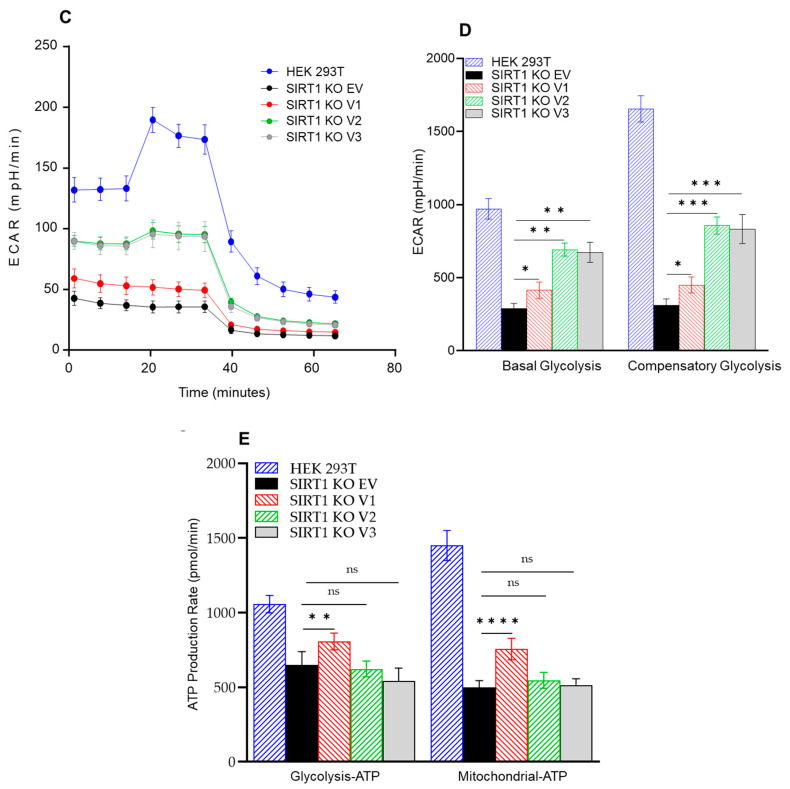
The effect of SIRT1 isoforms on mitochondrial function after transfection. HEk293T SIRT1-KO cells were transfected with SIRT1 isoform plasmids and the mitochondrial respiratory profile of these isoforms was examined with the Seahorse XFe96 analyzer. (**A**) Oxygen consumption rate (OCR) measurements were obtained over time (min) using an extracellular flux analyzer, Seahorse XFe96. (**B**) Basal mitochondrial OCR of SIRT1 isoform-transfected KO cells was derived by subtracting non-mitochondrial OCR. Maximum OCR was stimulated by FCCP addition. The spare respiratory capacity was calculated as the difference between maximal and basal OCR. (**C**) Glycolytic rate (ECAR) of SIRT1 isoform-transfected KO cells. (**D**) A quantitative analysis of the basal and compensatory glycolysis. (**E**) Quantification of total ATP production rate of KO cells transfected with different SIRT1 isoforms. Graphs represent data normalized with equal cell count, *n* = 3, one-way ANOVA, Tukey’s post hoc test (ns, *p* > 0.05, * *p* < 0.05; ** *p* < 0.01; *** *p* < 0.001; **** *p* < 0.0001).

To define the effect of the SIRT1 isoforms on cellular respiration without the interference of endogenous SIRT1 protein, the SIRT1-knockout cell line (SIRT1-KO) was used for the transfection and functional analyses. After the SIRT1-KO cells were transfected with the SIRT1-v1, v2, and v3 constructs, the cells were subjected to the Seahorse XF Cell Mito Stress Test and Glycolytic Rate Assay. The wild-type HEK293T cells had the highest basal respiration rate, the SIRT1-KO cells had the lowest basal respiration rate, but v1, v2, and v3 were able to increase the basal respiration rate significantly (Figure 7A,B). The deletion of SIRT1 reduced the maximal respiration rate, but all three isoforms were able to increase the maximal respiration rate (Figure 7A,B). The deletion of SIRT1 also reduced spare respiration capacity, but only the v1 isoform was able to significantly increase the spare respiration capacity (Figure 7A,B). Deletion of SIRT1 also reduced the cellular glycolysis; however, all three SIRT1 isoforms increased basal glycolysis and compensatory glycolysis (Figure 7C,D). These data indicate that alternative splicing excluded part of the N-terminal region in the SIRT1-v2 and v3 isoforms, which reduced their regulatory capability in the regulation of mitochondrial function.

## 4. Discussion

In the present study, we examined the impact of alternative splicing on the human SIRT1 gene, and the functional difference in regulating mitochondrial function among three isoforms, SIRT1-v1, v2, and v3, in mouse myocytes and human HEK293T cells.

The SIRT1-v1 isoform increased the mitochondrial membrane potential and upregulated the rates of mitochondrial oxygen consumption and ATP production. Moreover, SIRT1-v1 specifically upregulated complex I among the four complexes of the electron transport chain, which indicates that complex I is a direct regulatory target of the SIRT1 gene in the regulation of mitochondrial oxidative phosphorylation and ATP production. The targeted acetylation of lysine at histone H4 (K5, K8, K12, K16), and the gene expression of subunits of complex I, likely contribute to the overall impact of the SIRT1 gene on mitochondrial function in the myocytes. By contrast, the partial loss of the N-terminal domain attenuated the regulatory function in the v2 and v3 isoforms versus the v1 isoform.

Most mammalian genes undergo a series of mRNA processing steps before being translated into functional proteins [34]. Two splicing processes usually occur in cells. In the constitutive splicing process, the spliceosome removes intron(s) and ligates most exons in the order in which they appear in a gene [35]. In the alternative splicing process, certain splice sites are selected and certain exons are skipped; finally, the remaining exons are joined together to form a series of mRNA isoforms. As a result, different proportions of mature mRNA isoforms may be formed and found in cells and tissues [35], and the splicing isoforms tend to be tissue-specific and developmental stage-specific [36]. Further research is needed to explore the expression profile of SIRT1 isoforms and their role in various tissues during development, maturation, and aging.

The SIRT1 gene contains multiple exons. The full-length mRNA isoform (SIRT1-v1) is generated through the constitutive splicing process, whereas SIRT1-v2 and SIRT1-v3 are generated through alternative splicing. In addition, several other SIRT1 isoforms have been reported. For instance, the SIRT1-ΔExon8 isoform is the first reported SIRT1 splice isoform, in which exon 8 is skipped during alternative splicing [14]. Exon 8 is a part of the sirtuin catalytic domain, so loss of this exon causes the SIRT1-ΔExon8 isoform to have weak deacetylase activity. Multiple-exon skipping has also been observed in SIRT1-Δ2/9, in which the entire catalytic domain between exon 2 and exon 9 is lost due to splicing [37]. Several reports have analyzed the secondary structural elements of the sirtuin proteins and found 11 α helices (α1–α11) and nine beta (β1–β9) sheets within the sirtuin catalytic core domains among several sirtuin proteins, including SIRT1, SIRT2, and SIRT5 [38]. In the present study, the SIRT1-v1 isoform contains all the 11 alpha helices and nine beta sheets, but the SIRT1-v2 isoform lacks the alpha 1 helix and the beta 1 sheet, while the SIRT1-v3 isoform lacks the alpha 1, 2, and 3 helices and the beta 1 sheet.

It has been reported that the full-length SIRT1 protein contains two NLS (NLS1 and NLS2) as well as two NES (NES1 and NES2) sequences. Therefore, it has been observed to be shuttling between the nucleus and cytoplasm [39]. SIRT1 protein deacetylates not only nuclear proteins, but also cytoplasmic proteins, including acetyl-CoA synthetase [40] and cortactin [41]. In the present study, compared to the full-length SIRT1 protein (the same as SIRT1-v1 isoform), both the v2 and v3 isoforms lacked part of the N-terminal region that included nuclear localization sequences 1 and 2 (NLS1, NLS2), causing these two isoforms to be mainly localized in the cytoplasm. Therefore, the v2 and v3 isoforms might deacetylate the cytosolic proteins and have an unexpected impact on cellular metabolism and growth. It is possible that exclusion of the exon(s) in the C-terminal region in SIRT1 protein may change its catalytic activity and function as well.

Mitochondria are known to produce the majority of cellular energy in the form of ATP for normal cell function through oxidative phosphorylation [42]. Oxidative phosphorylation involves the electron transport chain (ETC) complexes, complexes I, II, III, and IV, and ATP synthase. The mitochondrial membrane potential (Δψm) serves as an intermediate form of energy storage which is used by ATP synthase to make ATP [43]. The mitochondrial membrane potential is an indicator used for assessing the process of electron transport and oxidative phosphorylation, and changes in the intracellular ATP level. In the present study, the SIRT1-v1 isoform increased the mitochondrial membrane potential (MMP) and upregulated the complex I activity. These data indicate that complex I is a direct target of the SIRT1 gene in the regulation of cellular metabolism.

Lysine acetylation is a reversible, post-translational protein modification process that regulates various cellular signaling pathways in health and diseases [44]. The SIRT1 protein plays a crucial role in the reversible acetylation of histone and non-histone proteins. Histone acetylation and deacetylation play critical roles in the regulation of chromatin, modifying the architecture and structure of the chromatin to facilitate or repress the transcriptional regulation. One of the well-documented modifications of newly synthesized histone H4 protein is the acetylation on the lysine residues at positions 5, 8, 12, and 16 (K5, K8, K12, K16) on the N-terminus. Deacetylation appears to usually occur quickly after protein synthesis and during the chromatin assembling [45]. The domain loss in the SIRT1-v2 and SIRT1-v3 isoforms could change the site-specific lysine acetylation in the histone H4 protein, which might also impact the SIRT1 gene in the regulation of the signaling pathway associated with the mitochondrial membrane potential and oxidative respiration.

In general, alternative splicing is coupled with transcription because both processes are under the control of the RNA polymerase transcription machinery. Multiple factors could affect the processes of transcription and alternative splicing, thereby affecting the total RNA transcript landscape. For instance, the RNA POL II elongation speed impacts the selection of constitutive splicing versus the alternative splicing and affects the ratio of the constitutively versus alternatively spliced isoforms [8]. Alternative splicing is also affected by the splicing factors that recognize and bind to specific target sequences located in exons or introns [1,5]. Additionally, histone acetylation changes the chromatin structure, which promotes splicing factors’ recruitment to a strong splice site that results in exon exclusion, or to a weak splice site that leads to exon inclusion [46,47]. It should be noted that SIRT1 isoforms could affect histone H4 acetylation and could potentially impact the coupled processes of transcription and alternative splicing of the SIRT1 target genes.

Analysis of deleted gene mutants has been widely used and has provided much additional knowledge on gene function [48]. The alternatively spliced isoforms are mimics of deletion mutants that are perfect tools to study the gene function. Our data indicate that the N-terminal domain is required for the SIRT1 gene as a positive regulator of oxidative phosphorylation, especially ETC complex I. Since the splicing variants are known to increase with advancing age, the functional impact of age-related changes in transcription and alternative splicing warrant further investigation [7,8,49].

## 5. Conclusions

In this report, we utilized a skeletal muscle cell line C2C12 and an HEK293T SIRT1-knockout cell line to study the effect of the partial exclusion of the N-terminus of SIRT1 on the regulation of cellular function. We also employed both the Seahorse XFe96 Analyzer and Oroboros oxygraph-O2K high-resolution respirometer to study the mitochondrial function because the O2K respirometer could be used to analyze the electron transfer complexes I, II, III, and IV individually with the manufacturer’s protocol. Here, SIRT1-v1 was found to specifically upregulate mitochondrial respiratory complex I, without significantly affecting the activity of complexes II, III, and IV, while loss of the N-terminal domains in the SIRT1-v2 and SIRT1-v3 isoforms attenuated their function.

Additionally, the SIRT1-knockout cell line (SIRT1-KO) was used to define the effect of SIRT1 isoforms on cellular respiration without the interference of endogenous SIRT1 protein. Apparently, deletion of SIRT1 significantly reduced OCR in the KO cells, while no significant increase in compensatory glycolysis was observed in the KO cells; instead, ECAR was also decreased in the KO cells. SIRT1-v2 and v3 upregulated OCR and ECAR to a certain extent in the KO cells, indicating that partial loss of the N-terminus weakened their ability to increase oxidative phosphorylation and glycolysis.

**Figure 8:**
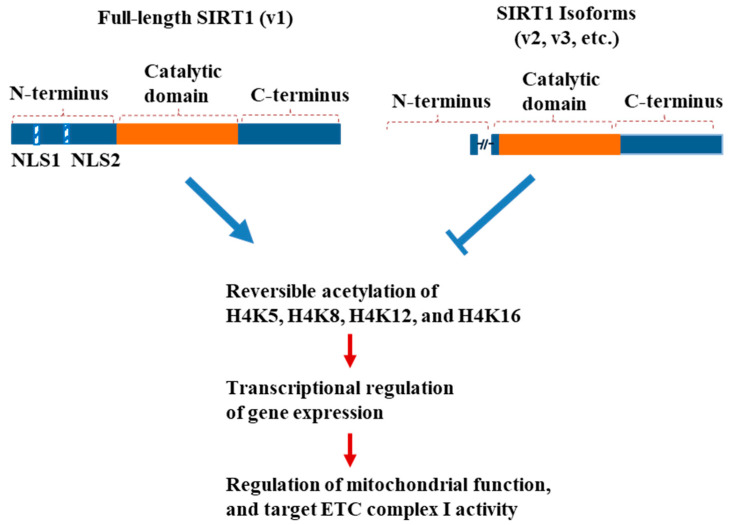
A proposed model of transcriptional regulation of mitochondrial function and ETC complex I by the SIRT1 isoforms. The partial exclusion of N-terminal region in SIRT1 gene modulates its regulatory capability in reversible acetylation of lysine at specific position(s), transcriptional regulation of gene expression, regulation of mitochondrial function, and ETC complex I activity.

Furthermore, domain loss affected the regulation of site-specific lysine acetylation in the histone H4 protein, and the gene expression of the respiratory complex I subunit, as well as the metabolic balance of oxidative phosphorylation versus glycolysis (Figure 8).

## Data Availability

The raw data are available without reservation upon reasonable request.

## Supplementary Materials

The following supporting information can be downloaded at: https://www.mdpi.com/article/10.3390/cimb46080522/s1, Figure S1: Transfection efficiency of SIRT1 isoforms in C2C12 cells. Figure S2: Activity of mitochondrial electron transport chain complexes using the Oroboros High-Resolution Respirometer. Figure S3: High-Resolution respirometer analysis of SIRT1-v3 transfection.
