# Supplementary material for: The Role of Sirtuin-1 Isoforms in Regulating Mitochondrial Function
Source: Curr Issues Mol Biol. 2024 Aug 14;46(8):8835–51. doi: 10.3390/cimb46080522 (PMC11352618; doi:10.3390/cimb46080522)
Supplement: Supplementary file 1 [file cimb-46-00522-s001.zip › cimb-3144544-supplementary.pdf]

Article

The Role of Sirtuin-1 Isoforms in Regulating Mitochondrial Function

Pankaj Patyal, Fathima S. Ameer, Ambika Verma, Xiaomin Zhang, Gohar Azhar, Jyotsna Shrivastava, Shakshi Sharma, Rachel Zhang, Jeanne Y. Wei *

Supplementary Figures

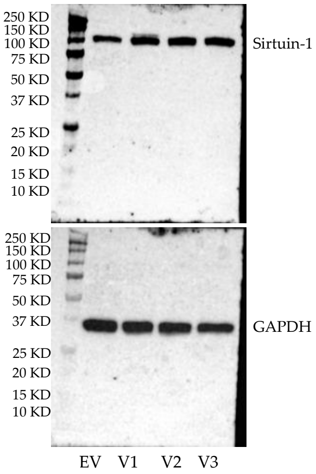

Figure S1. Transfection efficiency of SIRT1 isoforms in C2C12 cells. C2C12 cells were transfected with SIRT1 isoforms and western blot analysis were performed to test the efficenecy of transfection. Representative blot shows specific expression of SIRT1 isoforms after transfection and its respective loading control, GAPDH (n=3).

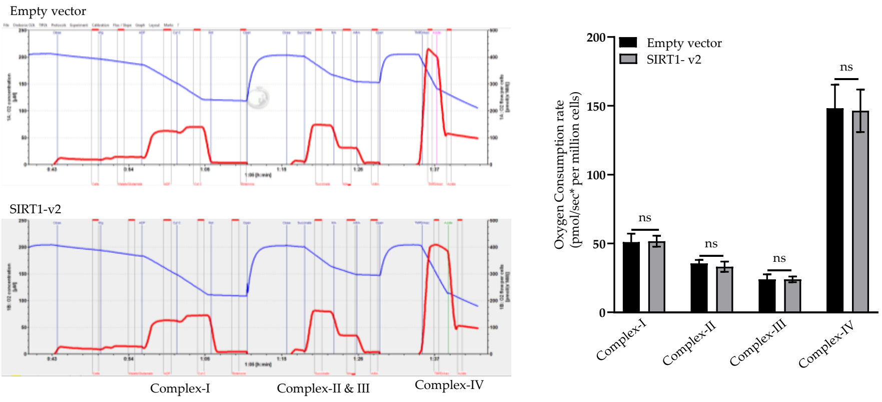

Figure S2. Activity of mitochondrial electron transport chain complexes using the Oroboros High-Resolution Respirometer. C2C12 cells transfected with SIRT1-v2 isoforms were permeabilized and were used to determine OCR level at complexes of ETC. Representative traces of the high-resolution respirometer were obtained using a multiple substrate-inhibitor-titration protocol. No change was observed in OCR levels of all complexes. Error bars represent mean SD, n=3. ns: $p > 0.05$.

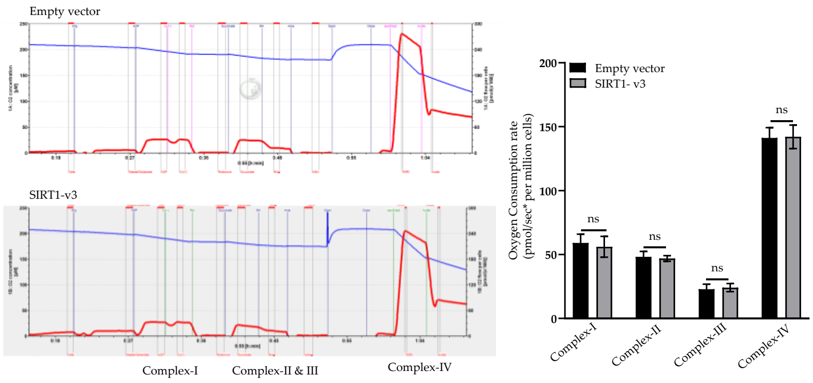

Figure S3. High-Resolution respirometer analysis of SIRT1-v3 transfection. C2C12 cells transfected with SIRT1-v3 isoform were permeabilized and were used to determine OCR level at complexes of ETC. Representative traces of the high-resolution respirometer were obtained and data analysis shows no change in OCR levels. Error bars represent mean SD, n=3. ns: $p >$ 0.05.
